# Phenotyping Root and Shoot Traits for Drought Response in Bambara Groundnut (*Vigna subterranea* (L.) Verdc.)

**DOI:** 10.3390/plants15081138

**Published:** 2026-04-08

**Authors:** Anne Linda Chisa, Takudzwa Mandizvo, Alfred Odindo, Paramu Mafongoya

**Affiliations:** 1 Crop Science, School of Agricultural, Earth and Environmental Sciences, College of Agriculture, Engineering and Science, University of KwaZulu-Natal, Private Bag X01, Scottsville, Pietermaritzburg 3201, South Africa; odindoa@ukzn.ac.za (A.O.); mafongoya@ukzn.ac.za (P.M.); 2 Centre for Transformative Agriculture and Food Systems, University of KwaZulu-Natal, Private Bag X01, Pietermaritzburg 3209, South Africa; takudzwamandizvo@gmail.com

**Keywords:** water scarcity, root system architecture, legumes, climate-smart agriculture, underutilised crops

## Abstract

Drought stress poses a significant challenge to food security in sub-Saharan Africa, particularly for smallholder farmers in dryland systems. Bambara groundnut (*Vigna subterranea* (L.) Verdc.), an underutilised legume with inherent drought tolerance, remains underexplored in terms of its root system traits. This greenhouse study investigated the early root and shoot responses of six Bambara groundnut genotypes under well-watered (100% field capacity) and water-stressed (50% field capacity) conditions using rhizotron-based phenotyping. Significant genotypic differences (*p* < 0.01) were observed in root traits such as root system depth (RSD: 11.0–19.9 cm), root system width (RSW: 6.96–12.2 cm), and root dry mass (RDM: 0.42–1.27 g). The ARC genotype exhibited a strong drought-avoidance strategy, increasing RSD from 12.2 to 19.9 cm and RDM from 0.42 to 1.16 g under stress. The Tiga Nicuru DIP-C-F7471 genotype showed adaptive plasticity, maintaining deeper roots (11.0–14.5 cm), high convex hull area (CHA), and root–shoot ratio (RSR) values, despite a reduction in RDM, suggesting a resource-conserving strategy. Principal Component Analysis (PCA) captured 93.6% of the total variability among genotypes. Root traits, particularly total root length (TRL), convex hull area (CHA), root system width (RSW), and root dry mass (RDM), were the main contributors to genotype differentiation. Strong positive correlations (r = 0.88–0.97) between root and shoot traits suggest that genotypes with more developed root systems also supported greater shoot growth, highlighting the coordinated response of above- and below-ground traits under drought stress. These findings provide valuable targets for breeding and highlight the value of rhizotron-based screening for root trait selection. Future field validation and full-season studies are recommended to confirm their relevance for improving yield stability in dryland agriculture.

## 1. Introduction

Crops frequently encounter a wide range of environmental stresses that jeopardise their survival, growth, and reproduction in natural ecosystems, while also negatively impacting crop yield and quality in agricultural farming systems [1,2]. Climate change, particularly drought and erratic rainfall, is an example of these stressors and continues to threaten food and nutritional security across sub-Saharan Africa [3].

Plants adopt three main strategies to cope with drought: avoidance, escape, and tolerance [4,5]. Drought avoidance involves morphological and physiological changes such as fewer stomata, smaller leaves, deeper roots, waxy cuticles, and leaf rolling to minimise water loss and osmotic stress [6,7]. Drought escape refers to rapid life cycle completion through early flowering, enhanced photosynthesis, higher nitrogen levels, and fast growth, allowing the plant to reproduce before drought onset [5,8]. While long crop cycles are ideal under normal conditions for maximum light capture, they reduce fitness under drought due to early soil moisture depletion [9,10]. Drought-tolerant plants accumulate osmolytes like proline and glycine, which activate the phenylpropanoid pathway, boosting lignin production and phytoalexin synthesis to stabilise membranes and resist stress [11,12,13].

Addressing water scarcity due to drought in agriculture is crucial for attaining Zero Hunger, one of the 17 Sustainable Development Goals outlined in the 2030 Agenda FAOSTAT [14]. Therefore, it is essential to develop high-yielding crops capable of thriving under limited water availability, especially in dryland farming systems, to support global food security [15]. Although considerable progress has been made in understanding how above-ground plant parts adapt to climate change, the study of root system responses has remained relatively overlooked

The root system is fundamental to the growth of terrestrial plants, serving as an anchor in the soil while also acting as the primary pathway for water and nutrient uptake from the surrounding environment [16,17]. Root system architecture (RSA) is the system that describes how roots grow, branch, and spread in the soil [18]. Traits such as root depth, lateral spread, and root-to-shoot allocation can influence a plant’s ability to survive and perform during water shortages [19]. Yet, root traits are often neglected in crop improvement research because they are harder to observe and measure than above-ground traits like plant height or yield [20]. This is especially true for underutilised crops like Bambara groundnut, where limited data on root development slows progress in identifying and breeding climate-resilient varieties.

Bambara groundnut (*Vigna subterranea* (L.) Verdc.) is highly drought-tolerant, nutritionally rich, and well-suited to low-input, smallholder farming systems [21]. However, despite its promise, Bambara groundnut remains under-researched, especially when it comes to understanding how its roots contribute to drought adaptation.

Some studies suggest that Bambara groundnut has a wide range of root growth patterns that differ between genotypes. Mayes et al. (2019) [21] found that the agroecological environment in which Bambara groundnut is grown, such as whether it originates from a dry or wet region, can influence root phenotypic variation. This highlights the importance of phenotyping root traits under water-limited conditions, as root architecture plays a critical role in drought adaptation. Given the increasing frequency of drought events, identifying landraces with superior root systems capable of accessing deeper or more persistent water sources is essential for improving resilience [22]. Variation in drought tolerance has been observed among Bambara groundnut landraces [18], underscoring the potential for selecting genotypes with enhanced performance under water stress.

Studying root traits, however, presents several methodological and logistical challenges that have historically limited progress in understanding below-ground adaptations. Root systems are complex and highly dynamic, making them difficult to measure without disrupting their natural growth environment [20]. Conventional methods are often destructive, labour-intensive, and time-consuming, providing only a snapshot of the root system at a given point rather than capturing its temporal development [23]. Moreover, field-based root studies are constrained by soil heterogeneity, environmental variability, and the difficulty of accessing deeper root layers, all of which complicate data interpretation [1]. These limitations have contributed to a significant knowledge gap in root phenotyping for many underutilised crops, including Bambara groundnut, where consistent and non-destructive methods for evaluating root system architecture (RSA) remain underdeveloped.

To address these challenges, rhizotron systems offer an effective approach. Rhizotrons are soil-filled, transparent-sided boxes or chambers that allow researchers to observe and measure root growth over time without disturbing the plant [24]. Unlike hydroponic systems or traditional destructive sampling methods, rhizotrons provide a more natural growing environment while still enabling detailed observation of root traits such as depth, angle, spread, and density. This makes them especially valuable for screening early-stage root development under different environmental conditions, such as water stress. Studies in other legumes (e.g., [25]) have shown that structures like rhizotron are reliable for capturing fine differences in RSA that are relevant for drought adaptation.

This study aims to address current knowledge gaps by assessing the root system architecture and early shoot growth of six Bambara groundnut genotypes, under both well-watered and water-stressed conditions using a rhizotron setup. The goal is to understand how these genotypes differ in their root development and allocation patterns in response to water availability. Identifying these differences is important for selecting Bambara groundnut varieties that are better suited to drought-prone areas. In the context of the climate crisis, this information can support smallholder farmers by improving access to crop varieties that perform better under dry conditions, while also helping plant breeders develop more resilient, locally adapted legume cultivars.

## 2. Results

### 2.1. Genotypic, Irrigation, and Interaction Effects on Root and Shoot Traits

Analysis of variance (Table 1) shows significant genotypic effects (*p* < 0.01) for several key root traits, including convex hull area (CHA), root branch count (RBC), root system depth (RSD), root system width (RSW), and total root length (TRL), highlighting substantial variability in root architectural traits among Bambara groundnut landraces. Genotypes also differed significantly (*p* < 0.05) in shoot length (SL) and leaf area (LA), reflecting above-ground morphological diversity. In contrast, genotype did not significantly influence biomass traits such as root dry mass (RDM), shoot dry mass (SDM), root mass ratio (RMR), shoot mass ratio (SMR), or root-shoot ratio (RSR), suggesting that dry matter partitioning was more stable across landraces.

**Figure 1:**
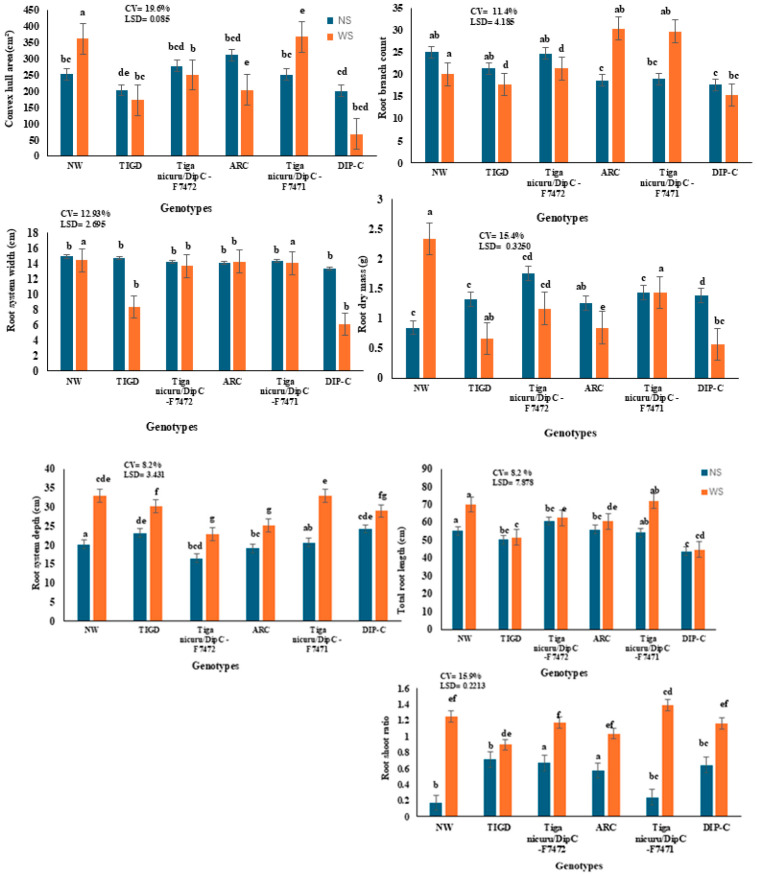
Comparative analysis of root morphological traits in six Bambara groundnut genotypes under no-stress (NS) and water-stressed (WS) conditions. Traits measured include convex hull area (cm^2^), root branch count, total root length (cm), root system width (cm), root dry mass (g), root-shoot ratio, and root system depth (cm). Plants were grown under two irrigation treatments: non-stressed (NS) and water-stressed (WS). Bars represent means ± standard error (n = 3). Different lowercase letters above bars indicate significant differences among genotype × irrigation combinations based on LSD at *p* ≤ 0.05. Coefficient of variation (CV%) and least significant difference (LSD) values are indicated for each trait.

Significant genotype × irrigation interactions (Figure 1) were detected for CHA (*p* < 0.01), RBC (*p* < 0.01), RDM (*p* < 0.01), RSD (*p* < 0.05), RSR (*p* < 0.01), RSW (*p* < 0.01), and TRL (*p* < 0.05), indicating that genotypic responses to water availability varied depending on the trait. Under WS conditions, the genotype Tiga Nicuru DIP-C-F7471 consistently maintained higher values for CHA, RDM, and RSR, while DIP-C and TIGD showed notable declines, suggesting contrasting drought responses. RSD increased in several genotypes under WS, particularly ARC and Tiga Nicuru DIP-C-F7471, highlighting their potential for improvement.

### 2.2. Principal Component Analysis (PCA) of Root and Shoot Traits

PCA was conducted to identify the major sources of variation among root and shoot traits in Bambara groundnut under contrasting water regimes. The first two principal components (PC1 and PC2) accounted for a cumulative 93.63% of the total variation, with PC1 explaining 72.33% and PC2 explaining 21.29%. PC1 (Table 2) was positively associated with key root architectural traits, including convex hull area (CHA, loading = 0.985), total root length (TRL, 0.991), root system depth (RSD, 0.982), root system width (RSW, 0.992), and root branch count (RBC, 0.920), as well as above-ground traits such as shoot length (SL, 0.920) and shoot dry mass (SDM, 0.855). This indicates that PC1 captures overall plant structural vigour and root system expansion. Notably, trait co-variation was evident, as genotypes exhibiting high RSW also tended to show elevated values for TRL and RDM, highlighting the functional linkage between horizontal spread, root elongation, and biomass accumulation under water stress.

PC2 (Table 2) was defined primarily by traits related to biomass allocation. Root-shoot ratio (RSR, loading = 0.836) and root mass ratio (RMR, 0.774) contributed positively, while shoot mass ratio (SMR, −0.774) loaded negatively, indicating a trade-off between shoot and root investment in response to moisture availability.

**Figure 2:**
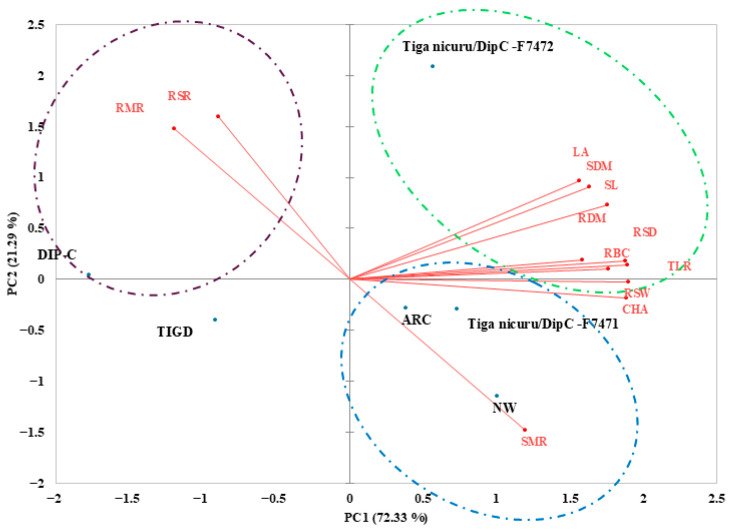
Principal component (PC) biplots demonstrating the relationships among root traits of six Bambara genotypes (ARC, N0v4, 519, and NW) at different water regimes: no-stress (NS) and water-stressed (WS). Convex hull area (CHA), shoot length (SL), total root length (TRL), root branch count (RBC), root system depth (RSD), leaf area (LA), root dry mass (RDM), shoot dry mass (SDM), whole plant dry mass (WPDM), root shoot ratio (RSR), shoot mass ratio (SMR), total root volume (TRV), and root mass ratio (RMR).

The PCA biplot (Figure 2) revealed clear genotype separation across water regimes. Tiga Nicuru DIP-C-F7471 and ARC under water-stressed conditions were positioned along the positive axis of PC1, aligning with traits indicative of deep, wide, and well-branched root systems. In contrast, DIP-C and TIGD showed displacement toward SMR and away from root architectural traits, suggesting limited adaptation to water stress. These results reinforce the importance of integrated root traits such as RSW, RDM, and TRL as functionally co-expressed indicators of drought resilience.

Overall, PCA highlights the central role of root architectural plasticity in mediating drought adaptation in Bambara groundnut. Genotypes with strong loadings for PC1 traits under stress conditions, particularly those maintaining coordinated development of root width, depth, and dry matter, represent promising candidates for selection in dryland breeding programmes.

### 2.3. Pearson Correlation Analysis of Plant Traits

**Figure 3:**
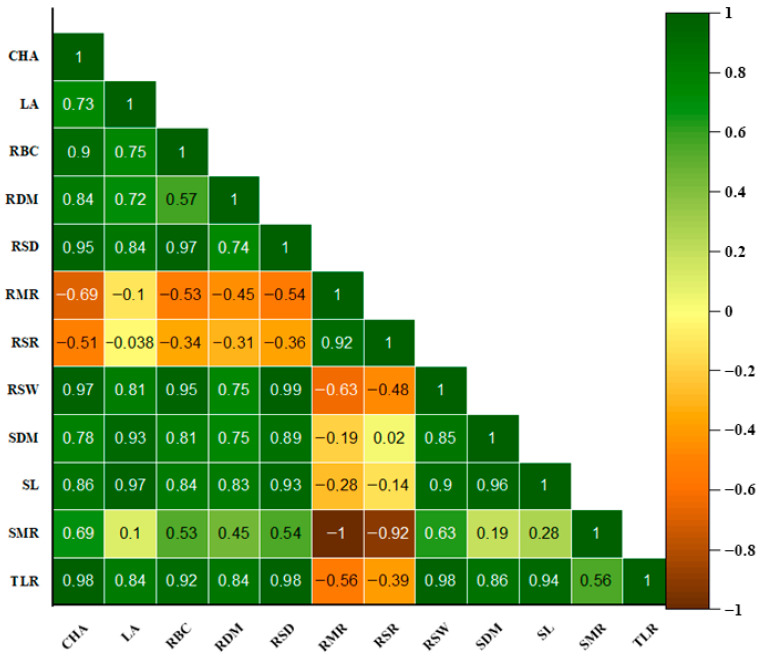
Pearson correlation matrix showing pairwise relationships among root and shoot traits of Bambara groundnut genotypes across irrigation treatments.

Pearson correlation analysis (Figure 3) revealed several strong and biologically meaningful associations among root and shoot traits, reinforcing the interconnected nature of drought adaptation in Bambara groundnut. Convex hull area (CHA) was highly correlated with total root length (TRL; r = 0.95), root branch count (RBC; r = 0.89), root system width (RSW; r = 0.97), and root dry mass (RDM; r = 0.88), indicating that genotypes with broader lateral root spread also tend to develop deeper, more branched, and heavier root systems. TRL also showed strong positive correlations with root system depth (RSD; r = 0.86), total root volume (TRV; r = 0.91), and RDM (r = 0.93), highlighting the functional coordination between root elongation, volume, and biomass accumulation under water stress.

Above-ground traits were positively linked to root development. Shoot length (SL) correlated strongly with CHA (r = 0.89), TRL (r = 0.90), and SDM (r = 0.84), suggesting that a well-developed root system supports enhanced shoot growth. Root mass ratio (RMR) and root-shoot ratio (RSR) were negatively correlated with shoot mass ratio (SMR; r = −0.77 and −0.72, respectively), reflecting the expected trade-off in biomass allocation under drought, where stress-resilient genotypes invest more in root growth at the expense of shoots.

Correlations were observed between convex hull area (CHA) and total root length (TRL), root branch count (RBC), root system width (RSW), and root dry mass (RDM), shoot length (SL), shoot dry mass (SDM), shoot mass ratio (SMR), root mass ratio (RMR), and root-shoot ratio (RSR). Darker shades indicate stronger correlations; only significant correlations (*p* < 0.05) are shown.

## 3. Discussion

Drought remains a dominant constraint to crop productivity in sub-Saharan Africa, with yield reductions of up to 50% in staple crops linked to increasing aridity and erratic rainfall patterns [26,27]. This study provides important insights into the drought adaptation responses of Bambara groundnut, revealing significant genotypic variation in root and shoot traits under contrasting irrigation regimes. These differences have direct relevance for breeding efforts targeting resilience in dryland agriculture.

Analysis of variance (Table 1) revealed significant genotype × irrigation interactions for key root traits, including convex hull area (CHA), root branch count (RBC), root system depth (RSD), root system width (RSW), root dry mass (RDM), and total root length (TRL). This highlights the presence of physiological plasticity, defined as the capacity of a genotype to adjust growth and allocation patterns in response to environmental stress [28]. As shown in Figure 1, ARC responded to water stress by increasing both rooting depth (12.2–19.9 cm) and root biomass (0.42–1.16 g), while maintaining a stable lateral spread. This reflects a coordinated drought-avoidance strategy that combines vertical root expansion with increased biomass allocation to enhance water uptake [29].

Tiga Nicuru DIP-C-F7471 also deepened its roots (11.0–14.5 cm) but exhibited a sharp decline in root system width (12.2–6.96 cm) and biomass (1.27–0.64 g), suggesting a more conservative strategy that prioritises accessing deeper moisture while limiting resource expenditure [30]. Developing deeper roots allows plants to reach water unavailable to shallow systems, and narrowing the root system can improve water uptake efficiency at lower metabolic cost [19]. Similar trends have been reported by [23], who found that crops with finer, deeper roots are better equipped to exploit subsoil moisture under drought conditions.

These contrasting patterns illustrate how genotypes express different drought-avoidance strategies, which may reflect a trade-off between acquisition efficiency and metabolic cost [4,5]. ARC’s deeper and denser root system aligns with high-input avoidance mechanisms, while DIP-C-F7471’s reduced biomass may indicate a more conservative strategy optimised for stress environments [6,7].

Some legumes, such as common bean, faba bean, and soybean, tend to reduce root growth under drought conditions [22,31,32], while chickpea shows genotype-specific responses depending on the type and timing of drought [33,34]. These interspecific differences further support the observed genotypic variation in Bambara groundnut and highlight the importance of screening for root traits when selecting for drought resilience.

In addition to root traits, Bambara groundnut genotypes differed significantly in shoot length (SL) and leaf area (LA) under water stress (Table 1), reflecting above-ground morphological diversity. This is consistent with previous reports on photoperiodic variation [35] and stress tolerance in this crop [36,37]. Although biomass-related traits (RDM, SDM, RMR, SMR, RSR) did not show significant genotypic effects under optimal conditions, RSD and RSR consistently increased under stress (Table 1), indicating biomass reallocation toward roots, a classic drought-avoidance mechanism [38]. Traits like CHA, RBC, and RDM remained relatively stable across treatments, suggesting they are environmentally robust and potentially useful as selection markers for drought tolerance.

Multivariate analysis confirmed these patterns. Principal Component 1 (PC1), which explained 72.3% of the total variation (Table 2; Figure 2), was driven by root traits (CHA, TRL, RSD, RSW, RBC) and SL. Genotypes such as ARC and DIP-C-F7471 grouped along PC1, indicating coordinated expression of root architectural traits under water-limited conditions. Similar results have been reported in chickpea [39] and bread wheat, where RSW was a major contributor to genotypic differences under drought [40]. The co-variation of RSW, TRL, and RDM observed in this study parallels findings in perennial grasses like *Lolium perenne,* where moisture availability controls horizontal and vertical root extension [41].

The PCA biplot also revealed clear genotype groupings based on their association with key root and shoot traits. The ARC genotype and Tiga Nicuru DIP-C-F7471 genotype clustered with root architectural traits such as root system width (RSW), total root length (TRL), convex hull area (CHA), and root system depth (RSD), indicating stronger root development linked to improved water acquisition under drought [25]. In contrast, DIP-C and TIGD grouped closer to shoot allocation traits, particularly shoot mass ratio (SMR), suggesting greater investment in above-ground growth [42]. These groupings highlight contrasting drought-response strategies among the genotypes and suggest that ARC and Tiga Nicuru DIP-C-F7471 may be promising candidates for selection in breeding programmes targeting drought-prone environments.

The observed variation in root architectural traits among Bambara groundnut genotypes provides important insight into potential drought adaptation strategies [43]. Root traits such as root system depth (RSD), root system width (RSW), and total root length (TRL) are key determinants of a plant’s ability to explore soil moisture under water-limited conditions [20]. Deeper root systems allow plants to access water stored in deeper soil layers, while greater lateral spread and root length increase the soil volume explored for water and nutrients [16]. These architectural adjustments are widely recognised as drought avoidance mechanisms, enabling plants to maintain water uptake even when surface soil moisture becomes depleted [24]. In this study, genotypes such as the **ARC** **genotype** and **Tiga Nicuru DIP-C-F7471 genotype** exhibited root configurations associated with improved soil exploration, including increased rooting depth and coordinated root expansion. Similar relationships between deeper or more extensive root systems and improved drought resilience have been reported in other legumes [25], where enhanced root architecture facilitates greater water capture and improved plant performance under water stress. These findings suggest that variation in root system architecture among Bambara groundnut genotypes may play a functional role in mediating drought adaptation by improving access to available soil moisture.

The Pearson correlation matrix (Figure 3) further clarified these trait interactions. CHA correlated strongly with TRL (r = 0.95), RBC (r = 0.89), RSW (r = 0.97), and RDM (r = 0.88), reinforcing the association between lateral spread and increased root depth, branching, and biomass. Similar relationships have been documented in maize [44], and ref. [45] also demonstrated that larger average root diameters lead to higher biomass accumulation. Positive associations between root traits and shoot performance (SL, SDM) observed here suggest that below-ground investment supports above-ground productivity under drought. SDM could serve as a useful proxy for root system performance, particularly in plant breeding programmes where large numbers of genotypes must be screened, and direct root phenotyping is costly or impractical.

## 4. Materials and Methods

### 4.1. Plant Material and Experimental Design

This experiment was conducted at the Controlled Environment Facility of the University of KwaZulu-Natal, Pietermaritzburg, Pietermaritzburg, South Africa (29°37′37.5″ S; 30°24′10.4″ E). Six Bambara groundnut (Table 3) were selected based on prior agronomic evaluation and visual distinction. Seeds were obtained from the University of KwaZulu-Natal’s Bambara groundnut germplasm collection. To ensure uniform germination and minimise variation due to environmental history, seeds were harvested during the same growing season and stored under controlled ambient conditions until use.

A completely randomised design (CRD) was used, arranged in a 2 × 6 factorial layout, comprising two water regimes (well-watered and water-stressed) and six genotypes. Each treatment combination was replicated three times, totalling 36 experimental units. To minimise spatial variation due to light, airflow, or temperature gradients in the greenhouse, rhizotron units were rotated every three days throughout the experimental period.

### 4.2. Greenhouse Conditions and Growth Medium

The experiment was conducted under controlled greenhouse conditions at the Controlled Environment Facility (CEF), University of KwaZulu-Natal, Pietermaritzburg, South Africa (29°37′37.5″ S, 30°24′10.4″ E). The mean air temperature was maintained at 25 ± 2 °C, with a relative humidity of 60 ± 3%, and a natural light photoperiod was supplemented to maintain 12 h of daylight.

The growth medium, comprised a mixture of Gromor Gromor (Pty) Ltd. (manufacturer, Umlaas Road, KwaZulu-Natal, South Africa) Potting Mix (30 dm^3^) and coarse sand in a 3:1 (*v*/*v*) ratio, was used as the growth substrate. This combination was selected following preliminary testing for optimal texture, drainage, and water-holding capacity, ensuring sufficient aeration while retaining enough moisture to simulate realistic soil conditions under both water-stressed and non-stressed treatments. The potting mix was sieved through a 2 mm mesh to remove debris, then sterilised at 105 °C for 24 h to eliminate microbial contaminants. The substrate was thoroughly homogenised and packed uniformly into each rhizotron to ensure consistent rooting conditions across treatments.

### 4.3. Rhizotron Construction and Setup

**Figure 4:**
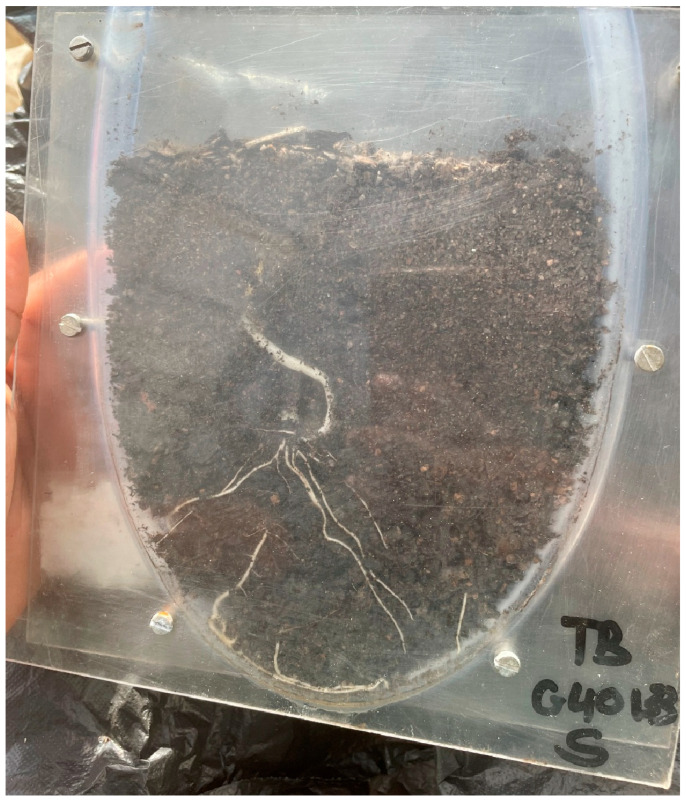
Rhizotron structure.

Rhizotron units were constructed following the design outlined by Mandizvo, Odindo [24], with minor modifications tailored to Bambara groundnut. Transparent acrylic sheets (3 mm thickness) were cut into rectangular panels measuring 30 cm in height, 20 cm in width, and with an internal depth of 3 cm. Plastic tubing (2 cm diameter) was cut to match the height and depth of the acrylic panels and served as lateral supports within the rhizotron structure (Figure 4).

To ensure precision during assembly, all components were aligned and clamped using a Grip GV9365 Bench Vice, South Africa (100 mm) before drilling pilot holes. The panels were then secured using Keystone Electronic machine screws (2.34 × 8.69 mm). Each assembled rhizotron enclosed approximately 1.8 × 10^−3^ m^3^ of soil and had a final weight of 0.95 ± 0.04 kg.

During plant growth, each rhizotron was positioned at a 45° angle to encourage root development along the transparent surface, facilitating non-destructive visualisation. To exclude light and simulate below-ground soil conditions, the transparent face of each rhizotron was covered with black polyethylene plastic throughout the experimental period.

### 4.4. Watering Treatments

Watering treatments were initiated at 10 days after sowing (DAS), once all seedlings had germinated and were visibly established. Two distinct soil moisture regimes were applied:

Well-watered (WW): Soil moisture maintained at 100% field capacity (FC)

Water-stressed (WS): Soil moisture maintained at 50% field capacity

Field capacity was determined gravimetrically by saturating a representative soil sample and allowing it to drain freely under gravity for 48 h. The difference between the saturated and oven-dried weights was used to calculate the water content at field capacity. Based on the rhizotron soil mass of 950 g per unit, the estimated water volume required to reach 100% FC was 145 mL. Accordingly, the water-stressed treatment received 70 mL to represent 50% FC.

The selection of 50% FC was guided by prior drought-screening protocols for legumes [25], which identified this threshold as sufficient to impose moderate but physiologically meaningful stress without inducing plant mortality. Preliminary calibration trials further confirmed that this level produced consistent reductions in growth and soil moisture content while maintaining plant viability throughout the experiment.

Rhizotrons were weighed daily using a digital scale, and water was replenished as needed using a graduated syringe to return each unit to its target weight. This method ensured consistent moisture levels per treatment while avoiding disturbance of the soil or root system.

### 4.5. Data Collection and Image Acquisition

Plants were harvested at 35 days after sowing (DAS) for data collection (Table 4). Shoots were cut at the soil line, and roots were carefully excavated for measurement. All harvested plant material was oven-dried at 60 °C for 72 h to determine dry mass.

**Figure 5:**
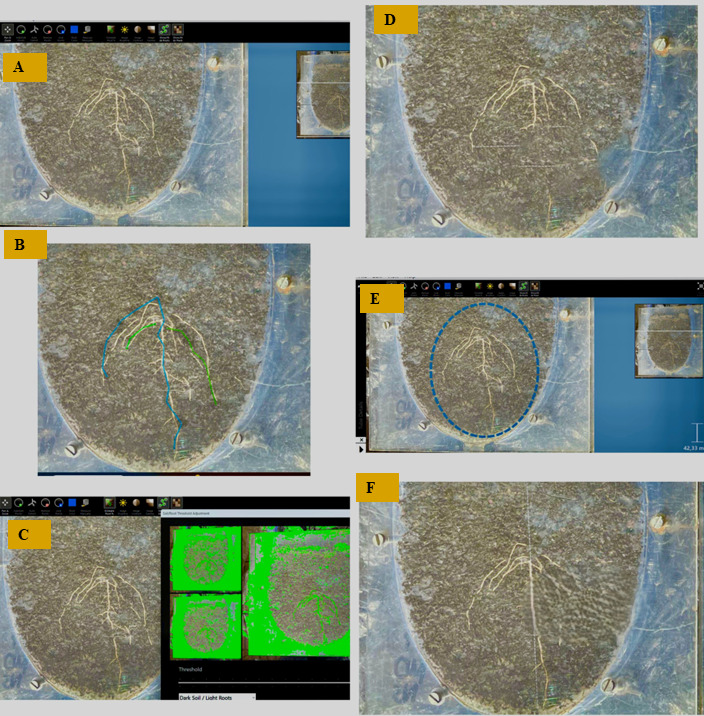
Illustration of how RootSnap software was used to analyse and collect data from captured root images: (**A**) root image in raw format imported from local storage to RootSnap; (**B**) tracing the root using Microsoft Surface Pro 4 stylus to measure total root length; (**C**) automated digital image analysis mode; (**D**) measurement of root system depth; (**E**) measurement of root convex hull area; (**F**) measurement of root system width.

Root growth was monitored through time-series image acquisition using a fixed camera setup. A digital camera (Apple iPhone 12, 16 MP, Apple Inc., Cupertino, CA, USA) was mounted at a consistent distance of 80 cm from the rhizotron. Images (Figure 5) were captured daily from both lateral sides of the rhizotron between 8 and 35 days after planting (DAP). The camera settings included a 4:3 resolution ratio, assistive grid enabled, a 3 s timer, and wide-angle mode (26 mm). Images were saved in raw format at a resolution of 3024 × 4032 pixels using the 12 MP wide camera.

Leaf area was measured using the Easy Leaf Area smartphone application (Heaslon, University of California, Davis, CA, USA).

### 4.6. Image Analysis

Root system 2-D images captured from the rhizotron were processed using RootSnap software (Version 1.3.2.25, CID Bio-Science Inc., Camas, WA, USA). The analysis was carried out primarily in automatic mode, with manual adjustments applied where necessary (Figure 5). A Microsoft Surface tablet, along with a Surface Pro 4 stylus, was used to manually trace and refine root outlines during analysis. Key architectural traits of the root system (as listed in Table 4) were extracted from these images using the semi-automated RootSnap software.

### 4.7. Statistical Analysis

All data were analysed using a two-way Analysis of Variance (ANOVA) in Genstat^®^ 22nd Edition (VSN International Ltd., Hemel Hempstead, UK) to assess the main effects of genotype, water regime, and their interaction on each measured root trait. The ANOVA was conducted under a factorial design, and treatment means were compared at a significance level of *p* < 0.05. When significant differences were detected, mean separation was performed using Duncan’s Multiple Range Test (DMRT) to identify statistically distinct groups. Mean values followed by the same letter were not significantly different, whereas those with different letters indicated significant differences. Before performing the ANOVA, data were tested for normality using the Shapiro–Wilk test and for homogeneity of variances using Levene’s test to ensure compliance with ANOVA assumptions.

To explore multivariate patterns and trait interrelationships, PCA was conducted using XLSTAT 2025 (Addinsoft, Paris, France) to identify key traits contributing to genotype differentiation under different water regimes. Additionally, Pearson’s correlation analysis was performed using OriginLab^®^ 2023 (OriginLab Corporation, Northampton, MA, USA) to assess relationships among root and shoot traits.

## 5. Conclusions

This study demonstrates that root system architecture plays a critical role in drought adaptation in Bambara groundnut. Significant variation among genotypes was observed in key root traits, particularly root system depth, root system width, total root length, convex hull area, and root dry mass, which were closely associated with improved plant performance under water stress. These traits represent important indicators of drought resilience and potential targets for selection in Bambara groundnut improvement programmes. Among the evaluated materials, the ARC genotype and Tiga Nicuru DIP-C-F7471 genotype showed promising responses under water-stressed conditions.

The successful application of rhizotron-based phenotyping in this study highlights its usefulness as a practical tool for early-stage screening of root traits in Bambara groundnut. Integrating root trait evaluation into breeding programmes can support the identification and development of drought-resilient genotypes, contributing to improved crop performance and resilience in dryland farming systems. Although the experimental scale was limited, it aligns with the constraints of controlled rhizotron-based phenotyping, which requires detailed monitoring of root development. Future research should incorporate larger genotype panels, field validation, and yield-based assessments to confirm the robustness and agronomic relevance of the identified traits.

## Figures and Tables

**Table 1:** Analysis of variance showing mean squares and significance levels for root and shoot traits of Bambara groundnut landraces under two water regimes.

Source of Variation	d.f	CHA	LA	RBC	RDM	RMR	RSD	RSR	RSW	SDM	SL	SMR	TRV	TRL
Genotype (G)	5	28,801 **	1071.4 **	58.778 **	0.447 **	0.003 NS	56.805 **	0.034 NS	23.243 **	0.283 NS	38.312 **	0.003 NS	0.0002 **	346.79 **
Irrigation (I)	1	59,278 NS	5490.8 **	16.000 NS	0.253 NS	0.197 **	610.337 **	3.789 **	54.072 **	2.430 **	180.231 **	0.197 **	0.001 **	428.08 **
G × I	24	16,732 **	99.3 NS	88.267 **	1.105 **	0.004 NS	17.680 *	0.217 **	16.928 **	0.170 NS	0.485 NS	0.004 NS	0.0006 NS	83.05 *
Residual	35	2263 NS	174.7 NS	6.167 NS	0.037 NS	0.0014 NS	4.146 NS	0.017 NS	2.557 NS	0.124 NS	5.646 NS	0.001 NS	0.0003 NS	21.85 NS

d.f; degrees of freedom, NS = not significant. * = *p* < 0.05; ** = *p* < 0.01. convex hull area (CHA), shoot length (SL), total root length (TRL), root branch count (RBC), root system depth (RSD), leaf area (LA), root dry mass (RDM), shoot dry mass (SDM), (root-shoot ratio (RSR), shoot mass ratio (SMR), total root volume (TRV), and root mass ratio (RMR).

**Table 2:** Summary of factor loadings, eigenvalue measure of sampling adequacy, percent, and cumulative variation for root traits.

Variables	PC1	PC2	PC3
CHA	0.985	−0.095	−0.099
LA	0.819	0.505	0.023
RBC	0.920	0.054	−0.069
RDM	0.827	0.098	−0.031
RMR	−0.625	0.774	−0.072
RSD	0.982	0.096	0.002
RSR	−0.466	0.836	0.016
RSW	0.992	−0.013	0.023
SDM	0.855	0.473	0.198
SL	0.920	0.380	−0.005
SMR	0.625	−0.774	0.072
TLR	0.991	0.072	−0.105
Eigenvalue	8.680	2.555	0.078
Variability (%)	72.332	21.294	0.647
Cumulative %	72.332	93.626	100.000

**Table 3:** List of selected Bambara groundnut genotypes.

Genotype	Colour	Origin
ARC	Brown	South Africa
Tiga nicuru/DipC-F7471	Red	Mali
Tiga nicuru/DipC-F7445	Purple	Mali
NW	Cream (white)	Namibia
Tiga nicuru/DipC-F7472	Brown (light)	Mali
DIP-C	Cream	Botswana

**Table 4:** Summary of the traits, definitions, methods, and units.

Trait	Full Name	Unit	Description
RSD	Root System Depth	cm	Vertical extent of the root system; primary measure of rooting depth
RSW	Root System Width	cm	Horizontal spread of the root system
CHA	Convex Hull Area	cm^2^	2D area occupied by the root system; approximated as a triangle (½ × RSW × RSD)
TRV	Total Root Volume	cm^3^	Estimated root system volume; calculated using a conical model
SRA	Seminal Root Angle	degrees (°)	Angle between outermost seminal roots; derived from RSW and FSRL geometry
SL	Shoot Length	cm	Length from base to tip of the shoot; reduced under water stress
LA	Leaf Area	cm^2^	Total leaf surface area; calculated from SL using empirical model
SDM	Shoot Dry Mass	g	Estimated dry mass of the shoot; based on LA
RDM	Root Dry Mass	g	Estimated dry mass of roots; based on TRV
RSR	Root-to-Shoot Ratio	ratio	Alternate expression of RSM; RDM divided by SDM
RMR	Root Mass Ratio	unitless	Proportion of total biomass allocated to roots: RDM/(RDM + SDM)
SMR	Shoot Mass Ratio	unitless	Proportion of total biomass allocated to shoots: SDM/(RDM + SDM)

## Data Availability

The data on the findings of this study can be obtained from the corresponding author upon reasonable request.
